# Facing death in care: Nurses’ lived experiences in the care of bedridden patients

**DOI:** 10.1017/S147895152510151X

**Published:** 2026-01-16

**Authors:** Ece Alagöz, Füsun Afşar

**Affiliations:** 1 Maltepe University, Istanbul, Turkey; 2 Yalova University, Yalova, Turkey

**Keywords:** Bedridden patients, caregivers, nurses, death awareness, end-of-life care

## Abstract

**Objective:**

This qualitative study explored nurses’ experiences of facing death while caring for bedridden patients in palliative and long-term care settings. Nurses are the primary witnesses to the final phase of life, where technical competence and emotional endurance coexist. Understanding how nurses perceive death and how knowledge, time, and communication affect their caregiving can provide insights into improving end-of-life nursing practices.

**Methods:**

The study was conducted with 70 primary nurse-caregivers of bedridden patients who were hospitalized in the palliative clinic of a university and an educational research hospital in Istanbul between April and August 2024. The research data were obtained through face-to-face interviews using a semi-structured interview form. The interviews were recorded on a voice recorder. The data obtained from the interviews were analysed thematically.

**Results:**

Three main themes were identified: Deficits in Knowledge and Education, Time Management, and Communication and Coordination. Nurses expressed uncertainty and emotional tension when providing care for dying patients. Inadequate end-of-life education heightened their fear of making mistakes. Heavy workload and limited time constrained emotional presence at the bedside. Fragmented communication among healthcare professionals increased feelings of isolation and moral distress. Across these themes, nurses experienced a silent but persistent awareness of death that shaped their professional identity and coping strategies.

**Significance of Results:**

Nurses caring for bedridden patients constantly face death, balancing medical duties with human vulnerability. Including death education, emotional support, and effective interdisciplinary communication in nursing practice can improve nurses’ resilience and the quality of end-of-life care.

## Introduction

Today, both developed and developing countries are making significant progress in medicine, health, science, and technology. Advances in medical technology, early diagnosis and treatment, and rising living standards are resulting in fewer deaths from diseases; however, as the population ages, the number of people living with chronic diseases is increasing (Hacker 2024; Hill et al. 2024). In Türkiye, demographic aging has led to a growing care problem in old age, increasing the demand for long-term care and placing additional pressure on both formal healthcare systems and informal caregiving structures (Tufan et al. 2022). Chronic diseases are recognized as a major health problem in developed countries due to increased life expectancy, and they place a serious burden on national economies (Hacker 2024; Hill et al. 2024). In particular, disability and/or bed dependency associated with chronic disease mean individuals require assistance with daily activities, leading to a greater need for care services (Soontorn et al. 2020; Tanrıkulu and Dikmen 2024).

Individuals with significant health limitations may struggle to participate in daily activities and self-care. As a result, they require ongoing assistance from family members or friends. These individuals are often called bedridden patients, and their family members or caregivers are known as caregivers. However, in institutional care settings, nurses constitute the primary caregivers responsible for delivering continuous, complex, and end-of-life care to bedridden patients. Caregivers of bedridden patients play a vital role in the healthcare system because many patients receive limited professional support from health institutions and nurses (Ceccon et al. 2021). Therefore, understanding the lived experiences of nurses caring for bedridden patients is essential for improving the quality and coordination of healthcare services.

Studies on bedridden patients show that nursing care is crucial for patient recovery and rehabilitation (Bhatt et al. 2024; Murayama et al. 2024; Santos de Carvalho et al. 2024). However, caring for a bedridden patient brings various challenges for nurses. Most of the problems faced by caregivers are similar regardless of the patient’s medical condition. Analyzing the challenges experienced due to care dependency can help identify common issues across cases and clarify how caregivers cope with the situation. It can also illuminate nurses’ expectations of nurses and what nursing care can do to support them. In this context, caregivers’ expectations not only shape the practical aspects of care but also influence the emotional, ethical, and professional experiences of nurses. This connection provides an essential foundation for understanding how nurses make sense of caregiving challenges and their encounters with death in daily practice.

As the population ages, the number of bedridden patients is increasing substantially. A bedridden patient often needs help with almost everything from basic activities such as eating, drinking, dressing, and toileting to more complex tasks, such as medication administration and monitoring vital signs (Yuan et al. 2024). This substantial responsibility typically falls on nurses, who are often family members. Evidence shows that caring for bedridden patients leads to significant physical and psychological stress for nurses (Noonan et al. 2018). Accordingly, understanding nurses’ expectations of nurses and the challenges they face is crucial for improving nursing care. A review of relevant literature indicates that caregivers encounter similar challenges despite differences in patients’ health status; common difficulties include limited understanding of illness, managing patients’ emotional outbursts, and inadequate utilization of health services when needed (Muhrodji et al. 2021; Simpson et al. 2021). Conversely, nurses’ expectations of nurses largely center on receiving appropriate information about the patient and practical guidance on coping with the situation.

Understanding expectations of nurses and the difficulties they face is important for developing effective solutions. Nurses are responsible both for delivering direct bedside care and for managing emotional, ethical, and practical aspects of care; therefore, all support directed to nurses ultimately enhances patient outcomes.

Nurses working with bedridden and terminal patients frequently encounter death and dying, which affects their emotional well-being, clinical decision-making, and communication behaviors. However, limited research has examined nurses’ lived experiences of facing death within palliative and long-term care settings.

Despite the growing body of research on care dependency, very few studies have examined how nurses experience and make sense of death while caring for bedridden patients. Existing studies primarily focus on caregiver burden, clinical care needs, or educational challenges, yet they rarely address the emotional and existential dimension of continual exposure to dying patients. The concept of death awareness nurses’ conscious recognition of the proximity of death and its influence on their clinical judgment, emotional state, and interpersonal interactions remains largely unexplored in palliative and long-term care contexts. Identifying this gap is essential because death awareness shapes nurses’ coping strategies, communication patterns, and quality of care. The present study seeks to address this gap by exploring nurses’ lived experiences of facing death in the daily care of bedridden patients.

## Purpose

This study aims to explore the lived experiences of nurses providing care to bedridden patients, with a particular focus on how they face death and how knowledge, time, and communication shape their caregiving practices.

## Methods

This study analyzed nurses ‘expectations of nurses and the challenges they face when caring for bedridden patients. A qualitative research design was used to explore nurses’ perceptions and experiences, considering the influence of social and cultural contexts. Qualitative research designs are particularly suitable for exploring individuals’ lived experiences, perceptions, and meanings within their social context (Aziz, 2014). In-depth interviews were conducted to gather detailed narratives from nurses. Ethical approval was obtained, and participants were informed about their rights and the purpose of the study. The sampling strategy involved purposive sampling, including caregivers from selected hospitals. Data collection took place in a private, quiet room within the palliative care units of the participating university hospital and the training and research hospital to ensure comfortable and confidential interviews. All interviews were conducted face to face and lasted approximately 25–45 minutes, depending on the participant’s willingness to elaborate on their experiences. Interviews were audio-recorded, transcribed, and translated for analysis, with key themes identified. Data collection took place in a quiet environment to ensure comfortable and confidential interviews. Interviews were audio-recorded, transcribed, and translated for analysis, with key themes identified. The qualitative framework effectively captured nurses’ narratives, highlighting their vital yet challenging role. Despite possible limitations and biases, the findings contribute to understanding nurses’ expectations and challenges (Hailu et al. 2024).

## Qualitative research approach

This study on nurses caring for bedridden patients uses a qualitative research approach. Qualitative methods are particularly suitable for exploring the personal experiences, emotions, expectations, and perspectives of caregivers, as they allow participants to describe their experiences in their own words (Onseng et al. 2024). Through rich and detailed narratives, qualitative research provides a deeper understanding of the subjective meanings caregivers attach to their caregiving roles and their interactions with nurses.

Data for this study were collected using in-depth, face-to-face individual interviews, which enabled open, flexible, and meaningful dialogue between the researchers and participants. These individual interviews provided a safe environment where caregivers could comfortably share sensitive and emotionally charged experiences related to providing care for bedridden patients. Building trust and rapport was essential to encourage participants to express their thoughts freely.

Qualitative methods were particularly appropriate for this study because they reveal both the emotional and practical dimensions of caregiving. This approach enabled the researchers to gain a nuanced understanding of caregivers’ expectations of nurses, the challenges they encounter, and the broader context of dependency and illness that shapes their experiences.

## Universe and sample

The caregiving needs of bedridden patients represent a growing public health concern due to increasing life expectancy and higher rates of chronic illness. As dependency levels rise, families experience greater physical, emotional, and social burdens, which increases the need for structured nursing support. Understanding how caregivers manage these responsibilities and what they expect from nurses provides essential insight into improving the quality and continuity of care.

Within this broad context, the present study focuses specifically on the primary caregivers of bedridden patients receiving care in palliative units. The study population consisted of caregivers of patients hospitalized in the palliative care units of a university hospital and a training and research hospital in Istanbul between April and August 2024. Purposive sampling was used to identify participants who could provide rich, relevant information regarding their caregiving experiences.

To capture a wide range of perspectives, maximum variation sampling was applied, enabling the inclusion of nurses with diverse demographic and caregiving backgrounds. A total of 74 caregivers were initially recruited; data collection continued until thematic saturation was reached, resulting in 70 nurses being included in the final analysis.

## Inclusion criteria

Nurses who undertook the role of primary caregiver for at least 1 month and who were over 18 years of age of the bedridden patient being cared for were included in the study. Primary caregivers with psychological or physical disorders, visual and hearing impairment were excluded from the study. Psychological distress was evaluated using a self-report screening item included in the demographic data form. Participants were asked the following question: *“Have you experienced any psychological distress (such as anxiety, stress, sadness, or emotional strain) in the past month?”* Response options were Yes or No. Participants who indicated *severe* psychological distress or ongoing psychiatric treatment were excluded. Mild or moderate self-reported distress was not an exclusion criterion.

## Data collection tools

Before starting the study, the open-ended questions were formed in a semi-structured form in order to create a general profile of the participants and to determine their characteristics that may be useful for future research. The interview guide is based on two main questions reported by the researcher: “Please tell us about your experiences while caring for your patient,” “What are your expectations from the nurses in the institution where you receive health care during this process?”

The semi-structured interview guide used in this study was specifically developed for this research. It was designed to explore the expectations and challenges caregivers of bedridden patients face in nursing care. The interview questions were created based on a literature review and expert opinions in the field. The Data Collection Form included demographic items (age, gender, marital status, education level, years of caregiving/nursing experience, current unit) and a self-reported psychological distress question used for eligibility screening.

## Data analysis

Data from all interviews were analyzed to provide an in-depth understanding of nurses’ lived experiences of caring for bedridden patients. The Consolidated Criteria for Reporting Qualitative Research (COREQ) checklist guided the methodological reporting process (Tong et al. 2007). Data saturation determined the final sample size, and interviews continued until no new themes emerged.

All audio-recorded interviews were transcribed verbatim and reviewed multiple times to gain familiarity with the data. Two researchers independently conducted initial coding. Early coding drew on conventional content analysis techniques to capture meaningful units within the narratives. Codes were then organized and compared to ensure consistency, and discrepancies were resolved through consensus. The coded data were exported to Excel to summarize code frequencies, track analytical decisions, and document non-confirming cases.

Following initial coding, the researchers proceeded with a phenomenological approach based on inductive thematic analysis, which was the primary analytical framework. This approach allowed themes to emerge directly from participants’ narratives without predefined coding structures. The thematic analysis process followed the five established steps:

1. Familiarization with the dataset,
2. Systematic coding,
3. Generating initial themes,
4. Reviewing and refining themes, and
5. Defining and naming final themes (Sundler et al. 2019).

A thematic map was constructed to illustrate relationships among codes and themes. To enhance the credibility and trustworthiness of the analysis, several rigor strategies were implemented, including peer debriefing with expert qualitative researchers and member-check interviews with selected participants to confirm the accuracy of interpretations.

As a result of this analytic process, three themes were identified: *Deficits in Knowledge and Training, Communication and Coordination*, and *Time Management*. Selected quotations were embedded to represent nurses’ experiences and provide thick description.

## Results

The demographic characteristics of the nurses (*n* = 70) are presented in Table 1.

**Table 1.** Demographic characteristics of nurses (*n* = 70)

Variable	*n*	%
**Age (years)**	Mean ± SD: **38.2 ± 8.9**	–
**Gender**		
Female	49	70.0
Male	21	30.0
**Marital status**		
Single	24	34.3
Married	46	65.7
**Education level**		
Primary/Secondary	18	25.7
High School	22	31.4
Undergraduate or above	30	42.9
**Years of nursing experience**		
1–5 years	23	32.9
6–10 years	25	35.7
11 + years	22	31.4
**Education level**		
Bachelor’s degree in nursing	47	67.1
Master’s degree	17	24.3
Vocational Health/Associate Degree	6	8.6
**Psychological distress (self-reported)**		
Yes	70	100

Table 2 illustrates the progression from initial codes to subthemes and final themes, offering a clear overview of the analytical structure used in the thematic analysis. Providing this coding schema enhances methodological transparency and strengthens the credibility of the findings.

1. Main Theme: Knowledge and Education

**Table 2.** Schematic distribution of qualitative themes and codes

Main themes	Sub-themes/Codes	Frequency (*n*)	% (out of 70)
**1. Knowledge and Education**	Lack of disease-related information	38	54.3
	Inadequate training in care procedures	35	50.0
	Fear of making a mistake	29	41.4
	Need for clear explanations from nurses	33	47.1
	Uncertainty about post-discharge care	31	44.3
**2. Time Management**	Feeling overwhelmed with care responsibilities	42	60.0
	Lack of personal time	30	42.9
	Needing nurses’ support to save time	27	38.6
	Physical and emotional fatigue	36	51.4
**3. Communication & Coordination**	Difficulty reaching physicians	39	55.7
	Nurses being too busy	34	48.6
	Fragmented care among healthcare professionals	32	45.7
	Expectation of empathy and tolerance	37	52.9
	One-way communication/lack of updates	33	47.1

In this study, nurses of bedridden patients emphasized the critical importance of addressing knowledge and educational gaps. These deficiencies in training appear to be at the center of many challenges nurses face and profoundly affect their ability to manage patient care effectively. During interviews, many nurses expressed feelings of inadequacy in their caregiving roles, as well as anxiety about how little they knew regarding their patients’ medical conditions and how this lack of understanding impacted their responsibilities.

In this study, carers listed many specific tasks expected of them when caring for a bedridden patient, but many felt unprepared for them. For example, one nurse stated, “It is difficult to feed him through the tube in his abdomen, sometimes it feels like it does not go away,” “We are constantly giving air, when his mask comes off, his oxygen drops,” a nurse of a patient with a home ventilator stated, “Being connected to a respirator is our biggest problem,” “I am afraid of doing something wrong to the patient.” In particular, they expressed their lack of knowledge about post-discharge medication management with the expressions “He takes a lot of medication, will we continue all of these medications at home?,” “What are the medications for?.” Many nurses also expressed their concerns about the post-discharge process, expressing their need for information as “”I am worried about what kind of process I will follow after discharge, “”I am waiting for them to explain what happened and what we need to do. These statements exemplify how a lack of understanding of the medical condition itself can leave carers feeling unprepared to address care. These gaps in knowledge of a patient’s condition and care practices can lead to increased stress for the nurse and poor outcomes for the patient. These statements suggest that targeted and individualized education of nurses about medical conditions and care practices is crucial. In addition to their lack of knowledge, nurses expressed their expectations in this regard as follows: “Nurse friends are more helpful. They teach care education visually and in detail, ‘Nurses direct care’, ‘I want them to explain the condition of my patient and what kind of process awaits us’.” In summary, the need for educational programs and resources for non-professional caregivers is highlighted as crucial to alleviate much of the stress and incapacity felt by nurses. Currently, existing educational programs are only available within the care services offered in hospitals. Nurses have a potential role to play in offering continuing education to caregivers. However, sufficient time and the right method of education are important. One caregiver said: “Sometimes I don’t understand, I don’t know what to do. I wait for them to help and tell me what to do at home. In this context, caregivers’ expressions of fear and uncertainty also reflected an underlying awareness of mortality. The anxiety of ‘doing something wrong’ was not only about harming the patient but also about the fear of losing them. This indicates that educational gaps are closely linked with emotional distress and death awareness. For caregivers, learning about medical procedures becomes a way to gain control in the face of potential loss, while nurses” educational guidance helps transform this fear into preparedness.

1. Main Theme: Time Management

A common struggle for nurses was managing time effectively. Despite their best intentions, many carers reported feeling overwhelmed by the demands of providing care while trying to fulfill their own responsibilities, such as work and personal care. Those who were unable to balance caring responsibilities with other life tasks felt that this had a serious impact on their mental health. Nurses used expressions such as “I have been here for days,” “I take care of my patient alone, I leave my patient to the nurses and go to do my job, but sometimes I wait because they are busy too,” “I do not have the strength and courage to care.”

As a result, many nurses indicated that a large part of their time management struggles depended on the availability of resources. Overall, this theme highlights the significant impact of time management issues on the overall well-being of nurses and suggests the need for more systematic support to alleviate these concerns.

Beyond the physical exhaustion, nurses’ struggle with time also represents a psychological confrontation with death. Time often feels suspended between hope and despair, as many describe days merging into nights around the patient’s bed. The passing of time becomes intertwined with the awareness of the patient’s decline, creating what can be described as a “rhythm of dying.” For nurses, each moment spent providing care carries both the value of presence and the silent anticipation of loss. Recognizing this temporal dimension of dying could guide nurses in offering emotional as well as practical support.

1. Main Theme: Communication and Coordination

Communication and coordination between nurses and nurses are often fraught with difficulties and represent significant challenges for nurses. Through in-depth interviews, nurses explained that while receiving healthcare, they were required to communicate not only with nurses but also with multiple professionals, such as physicians, physiotherapists, and dietitians, which often left them feeling helpless and overwhelmed on behalf of their bedridden loved ones. Nurses’ statements revealed several barriers to effective communication. Many nurses express the lack of time of nurses and doctors as follows: “Doctors only come for visits, they have a lot of work to do, but we cannot reach them when we want to ask something,” “Nurses are very busy, but they still try to come immediately when we need,” “I can only see the doctor during visits”, “it is time for us.” “I want them to separate.” In fact, while these expressions also express fragmented care, they also show that communication is almost always one-way. This is because health professionals communicate with nurses only when they need something. Nurses expressed that another important expectation in terms of communication is empathy with the expressions “I expect them to empathize when approaching the patient and us,” “I expect them to communicate with us with a smile,” “I expect them to approach our patient with tolerance and empathy.”

Within these narratives, the absence of empathetic communication can also be understood as the absence of shared acknowledgment of death. Nurses often stand alone in their emotional experience, silently confronting mortality without professional validation. When nurses approach communication with genuine empathy, they not only improve coordination but also help nurses voice their grief and fear. In this sense, communication becomes more than an exchange of information; it becomes a human connection that recognizes both life and its approaching end.

## Discussion

Three themes were identified in the interviews with the nurses of bedridden patients: Information and Training, Time management, Communication, and Co-ordination.

Caring for bedridden patients presents various expectations and challenges in accessing nurses. These expectations and challenges vary according to the patient’s condition, the age and health status of the nurse, and the nature of the relationship between them. Seventy primary nurses of bedridden patients from 2 hospitals, a university hospital, and a training and research hospital were interviewed in detail to explore their expectations of nurses. Three significant challenges were identified and analyzed: lack of knowledge transfer and training, communication barriers, and time constraints. Among these, information needs related to caregiving were considered the most fundamental issue.

### Information and training

This study revealed that nurses caring for bedridden patients experience significant knowledge and training gaps, which shape their professional responsibilities, emotional burden, and decision-making processes. Nurses frequently expressed feelings of uncertainty when caring for patients with complex or terminal conditions. Previous research also supports our finding that insufficient knowledge leads nurses to feel unprepared, increasing the risk of care-related errors and contributing to elevated stress levels (Bahrami et al. 2014). Studies indicate that a lack of information becomes increasingly complex as nurses. Similarly, communication process for bedridden patients continues, particularly when new and unexpected challenges arise (Dammann et al. 2022).

In the literature, it has been stated that caregivers of bedridden patients seek simple, basic medical information to understand the patient’s condition and illness better and to build trust in the healthcare professionals who provide treatment (Kıssal et al. 2019). At the same time, caregivers of bedridden patients seek information about what they can do to promote their patients’ recovery and how they can provide appropriate care (Farmahini-Farahani et al. 2019; Jukic et al. 2017). Providing caregivers with sufficient information about the patient’s medical condition is essential to enable informed care decisions (Schaffler et al. 2019). In a study conducted in Turkey, it was found that caregivers’ knowledge levels increased and their perceived burden of care decreased following an educational intervention (Öğür et al. 2019). The concept of time is essential for both caregivers and nurses in managing the care process of bedridden patients.

### Time management

Time management emerged as a pervasive challenge. Nurses reported experiencing overwhelming workloads, difficulty balancing multiple clinical tasks, and insufficient time for meaningful interaction with patients and families.

Research indicates that caregivers who provide care for more than 14 hours per day experience approximately twice the level of care burden compared to those who spend less time on caregiving (Ahmad Zubaidi et al. 2020). One of the most prominent expectations of primary caregivers is that nurses allocate sufficient time for patient and caregiver support (Andersen et al. 2024). Furthermore, studies have shown that inadequate communication and coordination during transition planning create significant challenges for the continuity and quality of care (Carbone et al. 2022). Toscan et al. (2013) similarly reported that fragmented care, involving multiple professionals without coordinated communication, places the caregiver in the position of the sole connector a pattern also observed in our interviews. Beyond these structural and practical issues, the findings reveal that caregivers live under a constant but often implicit awareness of death. While death may not have been mentioned explicitly, caregivers’ experiences of waiting, of tasks that “must be done before something irreversible happens,” align with what has been identified in end-of-life caregiving literature as a “silent proximity to death” (Bijnsdorp et al. 2022). This awareness amplifies the burden of information deficit: it is not only about not knowing what to do, but fearing that lack of knowledge may hasten the patient’s decline or death.

Time management difficulties become more than scheduling conflicts they reflect what caregivers describe as living in the “rhythm of dying.” The literature shows that caregivers of patients with life-threatening illnesses experience increasing burden as death approaches and describe their experience as temporal distortion, where days stretch and responsibilities multiply (Bijnsdorp et al. 2022). In this way, the expectation that nurses “make time” is loaded with existential meaning: time given is time bought from the impending ending.

Beyond the emotional dimension of time, existing literature highlights that many caregiver support services primarily focus on reducing caregiver burden rather than improving the quality of patient care itself. This imbalance can inadvertently limit the support caregivers receive for managing complex care tasks. As noted by Jowsey et al. (2013), the extensive health-related activities undertaken by caregivers often consume substantial amounts of time, restricting their ability to perform other essential tasks and potentially affecting the overall quality of care. These findings parallel the present study’s results, in which caregivers reported significant time pressure and unmet needs for practical nursing guidance.

### Communication and coordination

Similarly, communication and coordination issues appear frequently among nurses. When nurses say that nurses or doctors have no time, or that they cannot reach them when they need help, they are communicating not only frustration with service delivery but also with the fact that no one is helping them to prepare for the inevitable. Studies of caregivers in home death contexts highlight that the feeling of security is linked to clear, empathetic communication and to acknowledgment of the dying process (Barlund et al. 2021). Incomplete coordination between professionals leaves caregivers as lone witnesses of decline, which magnifies their emotional isolation.

These insights imply that nursing and caregiver support programs should extend beyond delivering information, scheduling time, and improving coordination. They should incorporate components of death awareness: helping caregivers recognize the trajectory of decline, supporting them in processing the emotional weight of facing mortality, and facilitating preparation rather than only reaction. The literature supports the idea that caregiver death-preparedness is associated with better outcomes for both the caregiver and the patient (Wen et al. 2025). Additionally, integrating death-awareness modules and structured reflective supervision sessions in palliative care units may support nurses’ emotional resilience and improve end-of-life communication. These practice-oriented strategies complement, but do not overlap with, the future research directions presented below.

## Strengths and limitations

This study has several strengths. First, it includes a relatively large sample of nurses working directly with bedridden patients in palliative care, offering rich and diverse perspectives. Second, data collection was conducted through in-depth, face-to-face interviews, allowing participants to share detailed experiences. Third, the study followed the COREQ guidelines and used a rigorous thematic analysis process, enhancing methodological transparency and credibility. These strengths contribute to the originality and depth of the findings and distinguish this study from previous research.

A key strength of this study is that it provides a rich and in-depth understanding of nurses’ lived experiences of facing death while caring for bedridden patients. The use of a qualitative phenomenological design allowed nurses to express their emotional, ethical, and professional perspectives in their own words, offering insights that are rarely captured in quantitative research. The inclusion of participants from both a university hospital and a training and research hospital enhanced data diversity and contextual validity. The rigorous thematic analysis process, guided by COREQ criteria and peer review, strengthened the trustworthiness and credibility of the findings.

However, this study also has several limitations. The data were collected from 2 hospitals in Istanbul, which may limit the generalizability of the results to other cultural or institutional contexts. The self-reported nature of the interviews may have been influenced by social desirability bias, particularly when discussing sensitive topics such as death and emotional distress. In addition, although member checks and peer debriefing were conducted, the interpretation of death awareness themes may still reflect subjective influences of the researchers. Future research could include longitudinal or multi-site designs to explore how nurses’ experiences of death awareness evolve across different care settings and cultural backgrounds.

## Future research

Future studies should further explore the multidimensional nature of nurses’ death awareness and its impact on professional practice, moral distress, and emotional resilience. Longitudinal qualitative research could examine how repeated exposure to dying patients shapes nurses’ coping mechanisms and ethical decision-making over time. Comparative studies across different healthcare settings such as intensive care, oncology, and home care may reveal how institutional culture influences nurses’ ability to face death and sustain compassionate care.

Quantitative or mixed-methods designs could also be used to develop and validate measurement tools that assess nurses’ death awareness and related competencies. Additionally, intervention studies focusing on death education, reflective supervision, and interprofessional communication training may provide evidence-based strategies to strengthen nurses’ preparedness for end-of-life care.

## Conclusions

Three themes were identified in interviews with caregivers of bedridden patients: Knowledge gaps; Time management difficulties; Communication problems. These three themes emphasise the importance of systematically assessing the abilities of caregivers of bedridden patients at the beginning of healthcare provision and creating moments of contact between caregivers and nurses. It also emphasizes the importance of promoting open communication and strong relationships between caregivers and health professionals. Regular contact opportunities, information transfer to caregivers together with all health professionals caring for their patients are recommended changes. Despite these recommendations, it is acknowledged that the implementation process is complex and time-consuming and requires the cooperation of various stakeholders, especially healthcare organizations. Nurses are the key stakeholders in the delivery of health services. As a result, it is aimed to inform caregivers about the critical support systems necessary for them to feel empowered rather than overwhelmed, and nurses have the most critical role in ensuring interdisciplinary coordination.

## Data Availability

No/Not applicable (this manuscript does not report data generation or analysis).

## References

[ref1] Ahmad Zubaidi ZS, Ariffin F, Oun CTC, et al. (2020) Caregiver burden among informal caregivers in the largest specialized palliative care unit in Malaysia: A cross sectional study. *BMC Palliative Care* 19(1), 186. doi:10.1186/s12904-020-00691-1 33292214 PMC7722979

[ref2] Andersen LH, Løfgren B, Skipper M, et al. (2024) “They forget that I’m a human being”-ward round communication with older patients living with frailty and informal caregivers: A qualitative study. *European Geriatric Medicine* 15(5), 1383–1392. doi:10.1007/s41999-024-01043-5 39227557 PMC11614924

[ref3] Aziz A (2014) *Research Methods and Techniques in Social Sciences*, 9th edn. Ankara: Nobel Academic Publishing.

[ref4] Bahrami M, Etemadifar S, Shahriari M, et al. (2014) 3 Informational needs and related problems of family caregivers of heart failure patients: A qualitative study. *Journal of Education and Health Promotion* 3, 113. doi:10.4103/2277-9531 25540786 PMC4275616

[ref5] Barlund A Sæle, André B, Sand K and Brenne A (2021) A qualitative study of bereaved family caregivers: feeling of security, facilitators and barriers for rural home care and death for persons with advanced cancer. BMC Palliat Care 20(1), doi:10.1186/s12904-020-00705-y PMC7796575 33419428

[ref6] Bhatt A, Kaur S, Dhandapani M, et al. (2024) Optimizing stroke care transitions: A patient-centered discharge program for caregivers. *Home Health Care Management & Practice* 37 (3), 191–201. doi:10.1177/10848223241282337

[ref7] Bijnsdorp F M, Onwuteaka-Philipsen B D, Boot C R, van der Beek A J and Pasman H Roeline. (2022). Caregiver’s burden at the end of life of their loved one: insights from a longitudinal qualitative study among working family caregivers. *BMC Palliat Care* 21(1). doi:10.1186/s12904-022-01031-1 PMC9364551 35945558

[ref8] Carbone S, Kokorelias KM, Berta W, et al. (2022) Stakeholder involvement in care transition planning for older adults and the factors guiding their decision-making: A scoping review. *BMJ Open* 12(6), e059446. doi:10.1136/bmjopen-2021-059446 PMC9196186 35697455

[ref9] Ceccon RF, Vieira LJES, Brasil CCP, et al. (2021) Aging and dependence in Brazil: Sociodemographic and care characteristics of older adults and caregivers. *Ciencia & Saude Coletiva* 26(1), 17–26. Portuguese, English. doi:10.1590/1413-81232020261.30352020. Epub 2020 Aug 33533838

[ref10] Dammann M, Staudacher S, Simon M, et al. (2022) 68 Insights into the challenges faced by chronically critically ill patients, their families and healthcare providers: An interpretive description. *Intensive and Critical Care Nursing* 68, 103135. doi: 10.1016/j.iccn.2021.103135 34736830

[ref11] Farmahini-Farahani M, Khankeh HR, Hosseini M, et al. (2019) Excruciating care: Experiences of care transition from hospital to home among the family caregivers of patients with spinal cord injury. *Nursing and Midwifery Studies* 10(1), 34–40.

[ref12] Hacker K (2024) The burden of chronic disease. *Mayo Clinic Proceedings: Innovations, Quality & Outcomes* 8(1), 112–119. doi:10.1016/j.mayocpiqo.2023.08.005 PMC10830426 38304166

[ref13] Hailu GN, Abdelkader M, Meles HA, et al. (2024) Understanding the support needs and challenges faced by family caregivers in the care of their older adults at home. a qualitative study. *Clinical Interventions in Aging* 19(14), 481–490. doi:10.2147/CIA.S451833 38500496 PMC10946444

[ref14] Hill AM, Starling T, Xin W, et al. (2024) Promoting healthy aging for older people living with chronic disease by implementing community health programs: A randomized controlled feasibility study. *International Journal of Environmental Research and Public Health* 21, 1667. doi:10.3390/ijerph21121667. 39767506 PMC11675327

[ref15] Jowsey T, McRae I, Gillespie J, et al. (2013) Time to care? Health of informal older carers and time spent on health related activities: An Australian survey. *BMC Public Health* 13(22), 374. doi:10.1186/1471-2458-13-374 23607727 PMC3641944

[ref16] Jukic PN, Gagliardi C, Fagnani D, et al. (2017) Home Enteral Nutrition therapy: Difficulties, satisfactions and support needs of caregivers assisting older patients. *Clinical Nutrition* 36(4), 1062–1067. doi:10.1016/j.clnu.2016.06.021 27461338

[ref17] Kıssal A, Kurt G, Koç M, et al. (2019) Knowledge and skill needs of home caregivers and their care burden perceptions. *Human Sciences* 16, 833–845. doi:10.14687/jhs.v16i3.5610

[ref18] Muhrodji P, Wicaksono HDA, Satiti S, et al. (2021) Roles and problems of stroke caregivers: A qualitative study in Yogyakarta. *Indonesia* 10(13), 380. doi:10.12688/f1000research.52135.2 PMC8822138 35186263

[ref19] Murayama LHV, Filho PTH, Winckler FC, et al. (2024) Caregiver burden, hopelessness, and anxiety: Association between sociodemographic and clinical profiles of patients with stroke. *Journal of Stroke and Cerebrovascular Diseases* 33(11), 107905. doi:10.1016/j.jstrokecerebrovasdis.2024.107905. 39103109

[ref20] Noonan MC, Wingham J and Taylor RS (2018) ‘Who cares?’ The experiences of caregivers of adults living with heart failure, chronic obstructive pulmonary disease and coronary artery disease: A mixed methods systematic review. *BMJ Open* 8(7), e020927. doi:10.1136/bmjopen-2017-020927 PMC6082485 29997137

[ref21] Öğür Z, Gözüm S, Taş E, et al. (2019) The influence of the education provided to family caregivers on bedridden patients and caregivers: A randomized controlled study. *Turkish Journal of Family Medicine and Primary Care* 13(3), 318–334. doi:10.21763/tjfmpc.446108

[ref22] Onseng P, Jiraporncharoen W, Moonkayaow S, et al. (2024) Expectation, attitude, and barriers to receiving telehomecare among caregivers of homebound or bedridden older adults: Qualitative study. *JMIR Aging* 7(7), e48132. doi:10.2196/48132. 38324373 PMC10882467

[ref23] Santos de Carvalho M, Souza Santos F and Bombarda Muller A (2024) Making the invisible visible: A characterization of bedridden and caregivers assigned to a Basic Health Unit. *Saúde E Pesquisa* 17(1), e–12035. doi:10.17765/2176-9206.2024v17n1.e12035

[ref24] Schaffler JL, Tremblay S, Laizner AM, et al. (2019) Developing education materials for caregivers of culturally and linguistically diverse patients: Insights from a qualitative analysis of caregivers’ needs, access and understanding of information. *Health Expectations* 22(3), 444–456. doi:10.1111/hex.12867 30767349 PMC6543161

[ref25] Simpson A, Bloom L, Fulop NJ, et al. (2021) How are patients with rare diseases and their carers in the UK impacted by the way care is coordinated? An exploratory qualitative interview study. *Orphanet Journal of Rare Diseases* 16(1), 76. doi:10.1186/s13023-020-01664-6 33568181 PMC7874609

[ref26] Soontorn T, Pongtriang P and Songwathana P (2020) Thai family caregivers’ experiences helping dependent elders during medical emergencies: A qualitative study. *Australasian Emergency Care* 23(2), 71–76. doi:10.1016/j.auec.2019.11.002 31926957

[ref27] Sundler AJ, Lindberg E, Nilsson C, et al. (2019) Qualitative thematic analysis based on descriptive phenomenology. *Nursing Open* 6(3), 733–739. doi:10.1002/nop2.275 31367394 PMC6650661

[ref28] Tanrıkulu F and Dikmen Y (2024) The effect on home caregivers of a family support program based on a nurse-led case management model: A randomized controlled pilot trial. *Home Health Care Management & Practice* 36(2), 102–111. doi:10.1177/10848223231205200

[ref29] Tong A, Sainsbury P and Craig J (2007) Consolidated criteria for reporting qualitative research (COREQ): A 32-item checklist for interviews and focus groups. *International Journal for Quality in Health Care* 19(6), 349–357. doi:10.1093/intqhc/mzm042 17872937

[ref30] Toscan J, Manderson B, Santi SM, et al. (2013) 13 “Just another fish in the pond”: The transitional care experience of a Hip fracture patient. *International Journal of Integrated Care* 13, e023. doi:10.5334/ijic.1103 23882170 PMC3718274

[ref31] Tufan İ, Tiryaki S, Doğan M, et al. (2022) The consequences of demographic developments in Türkiye and the care problem in old age. *Turkiye Klinikleri Journal of Gerontology* 1(1), 9–19. doi:10.5336/jgeront.2022-88300

[ref32] Wen B, Xie F, Ma J, Chen J, Liang L, Zeng Q, Ding J and Tang S (2025) Palliative care and its association with the quality of death in patients with advanced cancer in China: a cross-sectional study with a family caregivers’ perspective. *BMJ Open* 15(7), e081215. https://bmjopen.bmj.com/content/15/7/e081215 10.1136/bmjopen-2023-081215 PMC12248196 40645632

[ref33] Yuan L, Shen J, Ye S, et al. (2024) Assessing care dependence status and associated influencing factors among middle-aged hemiplegic stroke patients during the post-acute rehabilitation phase: A correlational study. *Journal of Clinical Nursing* 33(6), 2249–2258. doi:10.1111/jocn.17124 38509780

